# Time to Start a New Enhanced Recovery After Surgery (ERAS): A Retrospective Cohort Study

**DOI:** 10.7759/cureus.60301

**Published:** 2024-05-14

**Authors:** Ricardo Rodrigues, Jhonny Abreu, Beatriz Gonçalves, Mariana Luís, Catarina Freitas

**Affiliations:** 1 Department of Anaesthesiology, Hospital Central do Funchal, Funchal, PRT

**Keywords:** patient-centered approach, outcomes, enhanced recovery after surgery, perioperative care, colorectal surgery

## Abstract

Background: The enhanced recovery after surgery (ERAS®) is a multimodal perioperative care pathway designed to reduce surgical stress and ultimately improve patient recovery and outcome. It can require significant resources but with proven benefits. The main goal of this study was to perform a diagnostic assessment of perioperative practice in a local colorectal surgical center.

Methods: 93 patients who underwent elective colorectal surgery from January to December 2022 were analyzed. Preadmission, preoperative, and postoperative data of all patients were collected in a database developed by the researchers, according to ERAS® guidelines. Descriptive statistics were employed to summarize demographic and clinical characteristics. Chi-square and T-test were performed to identify possible associations between categorical variables and postoperative complications.

Results: Overall analysis showed deficient preoperative patient optimization, especially regarding nutritional counseling and supplementation, smoking and alcohol cessation, anemia treatment (9%), and pre-anesthetic medication (42%). Removal of invasive devices was significantly delayed (removal of urinary catheter average on the fourthday and surgical drain average on the fifth day) in the postoperatively period and oral intake (average onset on the sixth day). Both contribute to hospital length of stay (mean of 13 days) and a significant number of complications.

Conclusion: The results lead us to an individual and multidisciplinary reflection on current practices and outcomes. ERAS® program, already adopted by many centers, could have a positive impact on the immediate postoperative recovery of colorectal patients in Funchal Central Hospital and implementation seems necessary.

## Introduction

Enhanced Recovery After Surgery (ERAS®) is a perioperative multimodal program designed to facilitate early recovery after surgery by maintaining preoperative homeostasis and decreasing the physiological response to surgical stress [1]. ERAS® in colorectal surgery has been shown to decrease postoperative complications and consequently reduce hospital stay as well as morbidity, mortality, and readmissions compared to traditional methods of surgical care [2,3]. Surgical procedures entail significant healthcare costs, patient discomfort, and potential adverse outcomes. In response to this reality, the ERAS® program emerged from evidence-based practices with the aim of optimizing preoperative, intraoperative, and postoperative care.

The preoperative period involves counseling, multidisciplinary consultation, and specific nutritional requirements in case of major surgeries [4]. Intraoperative care includes strategies to reduce postoperative nausea and vomiting (PONV), multimodal analgesia, maintenance of body normothermia, and hydration [5]. Postoperatively, ERAS® emphasizes adequate fluid therapy, prevention of ileus, glycemic control, diuresis, nutrition, respiratory rehabilitation, and early mobilization [6]. These protocols also incorporate several specialized perioperative interventions that require a multidisciplinary team gathering surgeons, anesthesiologists, rehabilitation nurses, and nutritionists. The main goal, however, is to involve the patient in every stage of the process [7].

Despite its growing recognition and proven success, the introduction of ERAS® into our hospital reality has not yet been extensively explored. With this study, we intend to carry out a diagnostic assessment of colorectal surgical care in our local department, with the aim of identifying loopholes and facilitating the transfer to a standard of care based on the ERAS® program. We believe that the implementation of these practices by a health institution, as well as by its professionals, facilitates decisions about the best health care for patients. Reduction in hospital stay, improvement of the surgical experience, global satisfaction, and cost-efficiency ratio are recognized indicators of this program, which we aspire to adopt in our institution.

## Materials and methods

A retrospective cohort study was conducted on all patients submitted to elective major colorectal surgery between January and December 2022. The inclusion criteria were all patients undergoing elective major colorectal surgery, totaling 101 patients; eight patients were excluded for refusing to participate in the study so our final population was 93 patients.

The study was approved by the Funchal Central Hospital Ethics Committee (approval number 66/2023) and registered in ClinicalTrials.gov (ID S23005242). All participants completed and signed an informed consent form. Data was treated confidentially and collected from medical records after medical discharge. The researchers developed a data collection instrument considering the variables used in ERAS® 2018 guidelines for perioperative care in elective colorectal surgery [8]. Characterization data such as age, gender, and medical history were included.

Statistical analysis was performed using IBM SPSS Statistics for Windows, Version 26.0 (Released 2019; IBM Corp, Armonk, New York, United States). Demographic and clinical characteristics were summarized with descriptive statistics. Chi-square and T-test were performed to identify possible associations between categorical variables and postoperative complications.

## Results

Data from a cohort of 93 patients were studied. Demographic patient data and preoperative characterization are presented in Tables 1, 2. The mean age of the participants was 66 (range: 39-86), with the majority of these being male (49, 53%).

**Table 1:** Demographic patient data

	Mean (min-max)
Age (years)	66 (39-86)
Gender	n (%)
Male	49 (53)
Female	44 (47)

**Table 2:** Preoperative characteristics Hb, hemoglobin; BMI, body mass index; NRS, nutritional risk screening

	n (%)
Consultation	
Surgery/anesthesia	87 (94)
Nurse	0 (0)
BMI	
Underweight	1 (1)
Healthy weight	21 (30)
Overweight	37 (53)
Obese class 1	5 (7)
Obese class 2	6 (9)
NRS 2002	26 (28)
<3	13 (50)
≥3	13 (50)
Screening of anemia	
Hb <130 g/L	36 (39)
Male	13 (36)
Female	23 (64)
Anemia treatment	
Intravenous iron	5 (63)
Blood transfusion	2 (25)
Oral iron	1 (12)
Comorbidities	
Cardiovascular disease	64 (69)
Respiratory disease	13 (14)
Diabetes	25 (27)
Alcohol abuse	16 (17)
Avoided alcohol	7 (44)
Tobacco use	22 (24)
Smoking cessation	6 (27)
ASA performance score	
I	4 (4)
II	61 (66)
III	27 (29)
IV	1 (1)
Surgical procedure	
Hemicolectomy	38 (41)
Sigmoidectomy	23 (25)
Low anterior resection	22 (24)
Abdominal perineal resection	8 (8)
Colectomy	2 (2)
Neoadjuvant therapy	
Chemotherapy	3 (13)
Radiotherapy	1 (4)
Chemotherapy + radiotherapy	20 (83)
Surgical technique	
Laparoscopic	87 (94)
Laparotomy	6 (6)

The majority of patients (n=87, 94%, Table 2) had a preoperative surgical/anesthesia consultation. However, none of them had a nursing evaluation preoperatively. Only 26 (28%) of all patients were screened for nutrition risk using the NRS 2002 within the first 24 hours of admission. A Nutritional Risk Screening (NRS) score ≥3 identified a patient as nutritionally at risk, whereas an NRS score <3 indicated no nutritional risk. Our analysis revealed that 13 (50%) patients were at nutritional risk, presenting with an NRS score ≥3. Despite the results, no patient took nutritional supplements. Preoperative hemoglobin (Hb) <130 g/L was present in 36 (39%) out of 93 patients studied, and therefore, being considered anemic (ERAS Society 2018) [8]. Only eight patients underwent anemia treatment (five with intravenous iron infusions, two with blood transfusion, and one with oral iron therapy).

The prevalence of multimorbidity was high in patients presenting for colorectal surgery. The most frequent comorbidity was cardiovascular disease (69%) followed by diabetes mellitus (27%) and respiratory disease (14%). In addition, our study showed that according to the ASA classification, 61 (66%) patients had mild systemic disease (ASA II) and 27 (29%) had severe systemic disease (ASA III). Of the 93 cases analyzed, 22 (24%) were smokers and 16 (17%) had alcoholic habits. Despite this, 16 patients undergoing surgery continued to smoke during the perioperative period. Preoperative alcohol avoidance, for four or more weeks before surgery, was found in only seven patients.

In our hospital, the laparoscopic technique has been used in 87 (94%) of the surgeries. The most frequent surgical procedure performed was hemicolectomy in 38 (41%) patients, followed by sigmoidectomy in 23 (25%), low anterior resection in 22 (24%), abdominal perineal resection in 8 (8%), and total colectomy in 2 (2%).

Regarding the preoperative period, we took into consideration several strategies of optimization that are present in Table 3. Preoperative physiotherapy interventions were performed on 56 (60%) of the 93 patients by the rehabilitation nurse. In our cohort, bowel preparation was frequently used as well as prophylactic oral antibiotic therapy, being performed in 88 (95%) and 92 (99%) patients, respectively. The average fasting time was 11 hours in our study sample. In our clinical practice, we do not regularly administer oral carbohydrates preoperatively; the minimization of preoperative fasting was traditionally achieved through the administration of intravenous fluids. Within the total of 93 patients, fluids and electrolyte therapy was done in 40 (43%) patients and oral carbohydrates (CHO-maltodextrin 12.5%) was taken only by two (2%) patients. In this study, we report the use of benzodiazepines in 39 (42%) out of 93 patients, as the only preanesthetic medication.

**Table 3:** Preoperative optimization strategies

	n (%)
Respiratory physiotherapy	56 (60)
Antibiotic prophylaxis	92 (99)
Bowel preparation	88 (95)
Thromboprophylaxis	92 (99)
Sedative pre-medication	39 (42)
Fluids and electrolyte therapy	40 (43)
Carbohydrate loading	2 (2)

During the postoperative period (Table 4), 65 (70%) of the 93 patients did not have a nasogastric tube; when present, it was removed on average on the second day. The resumption of the oral diet in the postoperative period was done gradually. On average, fluid therapy was suspended on the fifth day.

**Table 4:** Postoperative factors

	n=93
Duration of IV fluid therapy, days - mean (min-max)	5 (2-22)
Nasogastric tube	
Yes - n (%)	28 (30)
No - n (%)	65 (70)
Time to removal of nasogastric tube, days - mean (min-max)	2 (1-11)
Urinary catheter	
Yes - n (%)	84 (90)
No - n (%)	9 (10)
Time to removal of urinary catheter, days - mean (min-max)	4 (0-30)
Surgical drains	
Yes - n (%)	77 (83)
No - n (%)	16 (17)
Time to removal of surgical drain, days - mean (min-max)	5 (2-21)
Early mobilization	
Yes - n (%)	57 (61)
No - n (%)	36 (39)
Opioid analgesia	
Yes - n (%)	70 (75)
No - n (%)	23 (25)

The Apfel score is a predictive tool used to assess the risk of PONV in patients undergoing surgery. According to Apfel score in our data, 76 (82%) patients had two or three risk factors for nausea and vomiting, and in these cases, two antiemetics were prescribed. Most of the patients (85%) received postoperative non-conventional analgesia. The practice of opioid use was high, being prescribed in 70 (75%) out of 93 patients.

In our cohort group, the removal of urinary catheters was performed on average on the fourth day (range: 0-30). Regarding the use of the surgical drain, there was a high prevalence; out of the total of 93 patients, only 16 (17%) did not have a drain postoperatively and its removal was on average on the fifth day (range: 2-21). Another factor studied in the postoperative period was early mobilization (early out-of-bed activities, especially ambulation on the first postoperative day). Among the 93 patients included in this study, early mobilization was performed on 57 (61%) patients. 

In this study, we found that the average time to the first bowel movement was on the third day, and the average time to start a solid diet was on the sixth day postoperatively (Table 5).

**Table 5:** Outcomes

	n=93
Lenght of stay, days - mean (min-max)	13 (4-67)
ICU admission - n (%)	5 (5%)
Time to first bowel movement, days - mean (min-max)	3 (0-7)
Time to solid oral intake, days - mean (min-max)	6 (2-17)
Reoperations - n (%)	6 (6%)
Readmissions - n (%)	10 (11%)
Deaths - n (%)	2 (2%)

In our cohort study, patients undergoing colonic surgery spent a median of 13 days in hospital. Additionally, we found that readmission after hospital discharge occurred in 10 (11%) patients in the studied population. When analyzing overall complications, the most frequent were surgical wound infection in 12 (13%) patients, postoperative ileus in 12 (13%), urinary infection in 12 (13%), cardiovascular complications in 9 (10%), and respiratory complications in 8 (9%) (Table 6).

**Table 6:** Postoperative complications

	n (%)
Surgical complications	
Surgical wound infection	12 (13)
Ileus	12 (13)
Wound dehiscence	1 (1)
Anastomotic dehiscence	3 (3)
Stoma complications	4 (4)
Medical complications	
Cardiovascular	9 (10)
Respiratory	8 (9)
Urinary	12 (13)

Although it was not the main aim of the study, we sought to correlate surgical and medical complications with some factors. No statistically significant correlation was found between the studied variables (Table 7).

**Table 7:** Factors associated with postoperative complications p<0.05 was considered statistically significant BMI, body mass index

Variables	X²	p	T-test
Surgical wound infection			
Diabetes	1.532	0.216	
Alcohol abuse	0.003	0.958	
Tobacco use	0.014	0.907	
Stoma complications	0.544	0.461	
BMI			0.633
Urinary complications			
Diabetes	1.532	0.216	
Duration of urinary catheter	0.082	0.774	
Gender	2.751	0.097	
BMI			0.481
Ileus			
Opioid analgesia	0.15	0.699	
Use of nasogastric tube	0.875	0.350	
BMI			0.577

## Discussion

This study presents a comprehensive analysis of the influence ERAS® protocols could have in improving surgical outcomes in our local unit, in all phases of perioperative care.

Regarding preadmission, several approaches can be optimized to enhance patient education and involvement. It is important to implement a multidisciplinary consultation with an anesthesiologist, nurse, and nutritionist, aiming at patient education through the surgical experience before hospital admission. Appropriate and complete information can reduce the anxiety associated with the entire surgical process, consequently reducing the need for premedication with benzodiazepines, which were overprescribed in the studied population, although highly unrecommended [9]. This assessment would also provide a better understanding of other anxiolytic approaches such as non-benzodiazepine drugs or the need for psychological education [10]. Patient-centered information contributes to greater patient satisfaction [11]. Timely preoperative consultation is also crucial for systemic disease optimization, as well as smoking cessation and avoidance of alcohol abuse. It is known that patients who smoke have an increased risk of intra and postoperative complications, and alcohol abuse increases postoperative morbidity, which is why smoking cessation and avoiding alcohol abuse are recommended four to eight weeks before surgery [12,13]. The assessment of nutritional status was insufficient and, as in our cohort, no patient received nutritional supplementation. Structured, multimodal prehabilitation protocols that include respiratory therapy and protein supplementation demonstrate a positive impact on preoperative physiological reserve with sustainable levels of functional capacity after surgery [14]. First, the rehabilitation approach was made by our nurses usually on the day before surgery. At this point, breathing exercise training was carried out and information regarding pre-surgical protocol was provided. This practice would be ideally managed in a timely preoperative consultation.

Regarding perioperative care, several aspects could be improved with ERAS® protocols, especially concerning patient comfort. Mechanical bowel preparation is widely used in our patients, causing dehydration, electrolyte disturbances, and discomfort to the patient [15]. This could be mitigated with a reduction of prolonged preoperative fasting and the provision of clear liquids with carbohydrates up to two hours before anesthetic induction [16]. It is recognized that these drinks improve general preoperative well-being, reduce postoperative insulin resistance, reduce protein loss, and maintain lean body mass and muscle strength [17].

Postoperatively, we found the presence of a nasogastric tube in 28 (30%) out of 93 patients. Studies show that removal before anesthetic reversal is associated with a lower number of respiratory infections, early return of intestinal motility, and greater tolerance to the oral diet [18]. The multimodal approach to nausea and vomiting prophylaxis should be considered in all patients using the Apfel scale. In the group studied (93 patients), 44 (47%) were female, 70(75%) used opioids, and 71 (76%) were non-smokers. These data corroborate the high percentage of risk factors for nausea and vomiting according to the scale applied. The prevention of PONV is essential in patients undergoing colorectal surgery, as its persistence is associated with dehydration, delay in starting the diet, nasogastric intubation, increased fluid therapy, increased hospitalization time, and increased costs with healthcare [19]. The high consumption of opioids is obviously not desirable nor recommended by the ERAS® protocol. The high need for analgesics observed in this cohort led us to a multidisciplinary reflection on the WHO recommendations for pain control [20]. We are aware that multimodal, opioid-sparing analgesia, started in the preoperative period, may contribute to better care in the future and this can be easily achieved with the correct involvement of the perioperative professionals, as defended by ERAS® policy. Removal of the bladder catheter is recommended between the first and third day after surgery. In this study, the catheter was removed on average on the fourth day (range: 0-30 days). This delay may increase the risk of urinary tract infection, delaying the patient's early mobilization. Intravenous therapy is usually not necessary the day after surgery for most of the patients undergoing colorectal surgery, but in this cohort, prolonged fluid administration was observed. Furthermore, a significant delay in restarting and progressing the diet was consistently seen. Both proved unnecessary but were simply being done because it is the established practice.

There are two important considerations with this research. The ERAS® protocol could be a useful tool for improving the surgical care provided to patients in our center, as corroborated by multiple studies [21]. In a healthcare system that wants to optimize its resources and improve the level of satisfaction of its patients, the implementation of the ERAS® protocols seems to be the way forward. On the other hand, the results lead us to an individual and multidisciplinary reflection. Defining new objectives aimed at a continuous quality improvement of provided care, particularly considering the current challenges, should be the direction to follow. The implementation of an ERAS® program will constitute a paradigm shift, with the possibility of developing new outcomes and new strategies in surgical care.

Limitations 

First, difficulty in accessing clinical information was noticed. Clinical records are kept in both paper and electronic formats, which makes data collection more challenging and time-consuming. Another limitation to be noted is the occultation bias or bias due to missing data, with the absence of some important perioperative variables, such as intraoperative data, surgical time, and blood loss, among others, which could have influenced the outcomes. In addition, a small sample also was a limitation of this study.

## Conclusions

This research provides strong suggestions that the implementation of an ERAS® program could bring several benefits and improved outcomes for patients undergoing major colorectal surgery in our local setting. It requires the commitment of all perioperative professionals, representing a change in the institution's culture but without major shifts from current practice. A mere change in protocols and mentalities, essentially through mass education, would lead to a significant, different, and optimized approach in a high-surgical-risk patient. ERAS® policy would enable and guide this multidisciplinary process.

The acknowledgment by the hospital's board of directors regarding the results of this study motivated the adoption of measures to improve the quality of medical and surgical care for patients undergoing colorectal surgery. As a first step, a multidisciplinary preoperative consultation was implemented, involving an anesthesiologist, nurse, and nutritionist, with the aim of optimizing patients proposed for surgery. Additionally, a training plan was developed for healthcare professionals involved in the care of colorectal patients, always based on the guidelines of the ERAS® program. Currently, we are in the negotiation phase for the acquisition of the ERAS® program. In the future, it would be highly pertinent to conduct a new study correlating ERAS protocol adherence and outcomes.
